# CORRELATION BETWEEN THE SEVERITY AND BLOOD ALCOHOL LEVEL OF TRAFFIC ACCIDENTS VICTIMS

**DOI:** 10.1590/1413-785220243201e271878

**Published:** 2024-05-06

**Authors:** Andressa Cruz Gonçalves, Henrique Silva Bombana, Alexandra Carolina Canonica, João Carlos Geber-Junior, Vilma Leyton, Julia Maria D’Andrea Greve

**Affiliations:** 1. Universidade de Sao Paulo, Faculdade de Medicina, Hospital das Clinicas HC-FMUSP, Programa Fisiopatologia Experimental, Sao Paulo, SP, Brazil.; 2. Universidade de Sao Paulo, Faculdade de Medicina, Departamento de Medicina Legal, Bioética, Medicina do Trabalho e Medicina Física e Reabilitação, Laboratorio de Toxicologia Forense, Sao Paulo, SP, Brazil.; 3. Universidade de Sao Paulo, Faculdade de Medicina, Departamento de Clínica Médica, Sao Paulo, SP, Brazil.; 4. Universidade de São Paulo, Faculdade de Medicina, Departamento de Ortopedia e Traumatologia, São Paulo, SP, Brazil.

**Keywords:** Ethanol, Wounds and Injuries, Accidents, Traffic, Injury Severity Score, Legislation, Blood Alcohol Content, Etanol, Ferimentos e Lesões, Acidentes de Trânsito, Escala de Gravidade do Ferimento, Legislação, Concentração Alcoólica no Sangue

## Abstract

**Objective:**

To evaluate the correlation between blood alcohol levels and the severity of injuries assessed by the Injury Severity Score (ISS) in patients who were victims of traffic accidents admitted to the Hospital das Clínicas of the Faculty of Medicine of the University of São Paulo (HCFMUSP).

**Methods:**

Cross-sectional study carried out between July 2018 and June 2019, at the Central Emergency Room of the Hospital das Clínicas of the Faculty of Medicine of the University of São Paulo (PSC-HCFMUSP). A total of 172 hospitalized patients victims of traffic accidents were included in this study. Blood samples were analyzed by the FMUSP Toxicology Laboratory.

**Results:**

36 patients (20.9%) had positive BAC (≥ 0.2 g/L) with a mean of 1.21 g/L. Overall, patients had a mean age of 37.2 years old, and 136 (79.1%) were men. The ISS of the total casuistry was 15.6; regarding the external cause, the motorcycle was ranked first with 100 cases (58.1%), and drivers were the majority with 57.4% of the sample.

**Conclusion:**

There was no correlation between the severity of the injuries and the blood alcohol levels of traffic accident victims admitted to a reference hospital. ***Level of Evidence II, Cross-Sectional Study.***

## RESUMO

**Objetivo:**

*Avaliar a correlação entre a alcoolemia e a gravidade das lesões avaliadas pelo Índice de Gravidade da Lesão (* Injury Severity Score* – ISS) em vítimas de acidentes de trânsito internadas no Hospital das Clínicas da Faculdade de Medicina da Universidade de São Paulo (HC-FMUSP).*

**Método:**

*Estudo transversal realizado entre julho de 2018 e junho de 2019, no Pronto Socorro Central do HC-FMUSP. Foram incluídas 172 vítimas de acidentes de trânsito. Amostras de sangue foram analisadas pelo Laboratório de Toxicologia da FMUSP.*

**Resultados:**

*36 pacientes (20,9%) apresentaram alcoolemia positiva (≥ 0,2 g/L), com valor médio de 1,21 g/L. No geral, os pacientes tinham uma idade média de 37,2 anos, e 136 (79,1%) eram homens. O ISS da casuística total foi 15,6; quanto à causa externa, a motocicleta ficou em primeiro lugar com 100 casos (58,1%); e os condutores foram prevalentes entre as vítimas (57,4%).*

**Conclusão:**

*Não houve correlação entre a gravidade das lesões e a alcoolemia das vítimas de acidente de trânsito internadas em um hospital de referência.* ***Nível de Evidência II, Estudo de Corte Transversal.***

## INTRODUCTION

 Traffic accidents are a global public health problem and closely associated with the consumption of alcohol. ^1^ In Brazil, according to Information Technology at the Service of SUS (DATASUS), there were 33,716 deaths caused by traffic accidents in 2020. ^2^

 In 2008, the government enacted Law No. 11,705 ^3^ called “ *Lei Seca* ” (Prohibition), establishing zero blood alcohol for drivers. This measure was attributed, among other factors, to the reduction in morbidity and mortality in the state and city of São Paulo related to traffic accidents. ^4^

 Alcohol consumption causes decreased visual ability, increased reaction time, impaired concentration and performance of tasks that require divided attention, in addition to an increased risk of collision. ^5^ However, there are controversies about the influence of alcohol on the severity of injuries caused by traffic accidents. ^6^^,^^7^ Tulloh and Collopy ^8^ report a positive association between alcohol intoxication, impact velocity, *Injury Severity Score* (ISS) and a higher risk of death. Other researchers claim that risky behavior (such as speeding and not wearing a seat belt or helmet) is not associated with the presence of alcohol in the blood. ^9^

There are many studies on the mortality of traffic accidents, while morbidity data are rarer, although important, due to the associated human and socioeconomic costs.

## MATERIALS AND METHODS

This is a cross-sectional study carried out between July 2018 and June 2019, at the Central Emergency Room of the Hospital das Clínicas of the Medical School of the University of São Paulo (PSC-HCFMUSP) in inpatients victim of traffic accidents. This study is part of the project entitled “Factors related to traffic accidents with victims treated at the Central Emergency Room and who were admitted to the Hospital das Clínicas of FMUSP”, approved by CAPPesq HCFMUSP (No. 2,071,227).

 A total of 172 patients were included, victims of traffic accidents who were admitted to the Central Emergency Room and were hospitalized at the Hospital das Clínicas of FMUSP. Blood samples from the victims were collected in *Vacutainer ®* tubes containing sodium fluoride and EDTA. The asepsis of the collection site was performed with non-alcoholic solution in order to avoid contamination. Samples were sent to the Toxicology Laboratory of the Department of Legal Medicine, Bioethics, Occupational Medicine and Physical Medicine and Rehabilitation of FMUSP for toxicological analysis by gas chromatography-FID after extraction via *headspace* , a methodology already established in the laboratory. For this study, the value adopted for positive blood alcohol levels was ≥ 0.2 g/L based on Law 11,705/2008. ^3^

 After the initial care and stabilization of the victim, the medical team and/or trained nurses applied the ISS through a tool built and adapted from CAIS 85–F and CAIS 85-P, maps that summarize the AIS manual and that give greater agility in the calculation of the ISS ^10^ already validated and implemented in the institution. In doubtful cases, consensus was established by team discussion. 

After referral to inpatient units (wards), conscious patients were approached to sign the Informed Consent Form, and a questionnaire was applied to obtain sociodemographic and accident-related information. For patients who were in a coma, intubated for more than 10 days or who could not answer the questions, the interview and signing of the ICF was done by a family member or legal guardian.

 For categorical variables, the results were presented as mean, standard deviation and frequencies (percentages). In the continuous variables, distribution analyses were performed. The relationship between the different ISS groups and the categorical variables was performed using Pearson’s chi-square test. The F–ANOVA test was used to identify deterministic relationships between a categorical variable and a continuous variable. The analyses were performed using the *R Core Team 2021 software* , and the significance level was established as p < 0.05. 

## RESULTS

During the collection period, 909 patients with traumatic injuries were treated at PSC–HCFMUSP. Of these, 172 patients were eligible for inclusion in the study.

The age of the patients ranged from 18 to 87 years old (37.2 ± 14.7) and 136 of them (79.1%) were men. The age of males (34.68 ± 10.7 years) was higher than that of females (20.5 ± 2.1 years) for the alcohol-positive group. The age of the female victims varied between the negative 47.38 ± 20.88 years versus positive 20.5 ± 2.12 years groups.

 Other sociodemographic information and the relationship between the categorical variables of the different groups can be seen in Table 1 . 

**Table 1.** Sociodemographic characteristics of the patients included in the study separated by Positive and Negative Group.

**Category**	**ALL n (%)**	**POSITIVE n (%)**	**NEGATIVE n (%)**	***p*** **= value**	**X²**
**Gender**				0.0298	4.7203
Male	136 (79.1%)	32 (23.5%)	104 (76.5%)		
Female	36 (20.9%)	2 (5.6%)	34 (94.4%)		
All	172	36 (20.9%)	138 (80.2%)		
**Age range**				0.233	5.5781
18 to 29	60 (34.9%)	14 (23.3)	46 (76.6%)		
30 to 39	52 (30.2%)	11 (21.2%)	41 (78.8%)		
40 to 49	32 (18.6%)	5 (15.6%)	27 (84,4%)		
50 to 59	13 (7.6%)	4 (30.8%)	9 (69.2%)		
≥ 60	15 (8.7%)	0 (0%)	15 (100%)		
**Education level**				0.6273	1.7436
Illiterate	0 (0%)	0 (0%)	0 (0%)		
Incomplete elementary school	37 (21.5%)	7 (18.9%)	30 (81.1%)		
Complete elementary school	14 (8.1%)	3 (21.4%)	11 (78.6%)		
Incomplete High school	28 (15.7%)	7 (25%)	21 (75.0%)		
Complete high school	61 (35.5%)	16 (26.2%)	45 (73.8%)		
Higher Education	29 (16.9%)	1 (3,4%)	28 (96.6%)		
N/C	3 (1.7%)	0 (0%)	3 (1.7%)		
**Skin color/race**				0.1415	6.8954
White	78 (45.3%)	13 (16.7%)	65 (83.3%)		
Mixed	63 (36.6%)	14 (22.2%)	49 (77.8%)		
Black	25 (14.5%)	6 (24%)	19 (76%)		
Asian	3 (1.7%)	0 (0%)	3 (100%)		
N/C	3 (1.7%)	1 (33%)	2 (67%)		
**Driver's License**				0.9507	0.0038
Yes	107 (62.2%)	21 (19.6%)	86 (80.4%)		
No	61 (35.5%)	13 (21.3%)	48 (78.7%)		
N/C	4 (2.3%)	2 (50%)	2 (50%)		

N: number of patients; %: percentage of patients; NC: not included

 In the total sample, 36 (20.9%) victims had positive blood alcohol (≥ 0.2 g/L), with a mean of 1.21 ± 0.75 g/L. The alcohol concentration in the positive Alcohol Group ranged from 0.3 to 2.8 g/L. Twenty-five (69.4%) patients had alcohol ≥ 0.6 g/L and presented ISS 16.48. Male victims had a mean blood alcohol level of 1.26 ± 0.75 g/L *versus* 0.39 ± 0.11 g/L for females. 

 Drivers, with 19 cases (20.2%), were prevalent among the alcohol positive group, in addition, motorcycle, with 100 cases (58.1%), was the vehicle most involved with ISS = 15.52 ± 11.23, a lower value compared to other vehicles 17.46 ± 12.25. There was no correlation between the variables type of victim, vehicle, type of accident, day of the week and type of care, and the degree of alcohol level. ( Table 2 ) 

**Table 2.** Characterization of the types of accidents suffered by the patients included in the study, separated into the Positive and Negative Blood Alcohol Content groups

**Category**	**ALL n (%)**	**POSITIVE n (%)**	**NEGATIVE n (%)**	***p*** **= value**	**X²**
**Victim**					
Driver	94 (54.7%)	19 (20.2%)	75 (79.8%)	0.7239	1.3222
Passenger	23 (13.4%)	6 (26.1%)	17 (73.9%)		
Pedestrian	46 (26.7%)	7 (15.2%)	39 (84.8%)		
Cyclist	8 (4.7%)	2 (25%)	6 (75%)		
N/C	1 (0.6%)	0 (0%)	1 (100%)		
**Vehicle**					
Motorcycle	100 (58.1%)	19 (19%)	81 (81%)	0.8033	1.6305
Automobile	32 (18.6%)	6 (18.8%)	26 (81.2%)		
Bicycle	9 (5.2%)	3 (33.3%)	6 (66.7%)		
Bus	11 (6.4%)	3 (27.3%)	8 (72.7%)		
Truck	13 (7.6%)	2 (15.4%)	11 (84.6%)		
N/C	7 (4.1%)	1 (14%)	6 (86%)		
**Type of Accident**					
Frontal collision	50 (29.1%)	9 (18%)	41 (82%)	0.2459	7.8952
Lateral collision	30 (17.4%)	10 (33.3%)	20 (66.7%)		
Rollover	5 (2.9%)	1 (20%)	4 (80%)		
Fall	12 (7%)	2 (16.7%)	10 (83.3%)		
Runover	50 (29.1%)	7 (14%)	43 (86%)		
Rear collision	7 (4.1%)	0 (0%)	7 (100%)		
Multiple	5 (2.9%)	0 (0%)	5 (100%)		
N/C	18 (10.5%)	5 (28%)	13 (72%)		
**Day of the Week**					
Monday	32 (18.6%)	8 (25.0%)	24 (75.0%)	0.1091	10.3909
Tuesday	18 (10.5%)	2 (11.1%)	16 (88.9%)		
Wednesday	26 (15.1%)	4 (15.4%)	22 (84.6%)		
Thursday	26 (15.1%)	2 (7.7%)	24 (92.3%)		
Friday	23 (13.4%)	4 (17.4%)	19 (82.6%)		
Saturday	25 (14.5%)	5 (20%)	20 (80%)		
Sunday	22 (12.8%)	9 (40.9%)	13 (59.1%)		
**Pre-hospital Care**					
Yes	160 (93%)	30 (18.8%)	130 (81.3%)	1.00	0.00
No	6 (3.5%)	1 (16.7%)	5 (83.3%)		
N/C	6 (3.5%)	3 (50%)	3 (50%)		
**Type of Service**					
SAMU	58 (33.7%)	10 (17.2%)	48 (82.8%)	0.9488	0.3578
Fire Department	31 (18%)	5 (16.1%)	26 (83.9%)		
Águia	61 (35.5%)	11 (18%)	50 (82%)		
Other	8 (4.7%)	2 (25%)	6 (75%)		

N: number of patients; %: percentage of patients; NC: not included; *X²:* chi-square; *P:* degree of significance.

* *P* -value < 0.05

 The ISS of the total sample was 15.6 ± 11.06 (p = 0.65), with no significant difference in male patients with negative and positive blood alcohol levels (p = 0.62). Women showed greater variation between the negative and positive groups (p = 0.08). The distribution of the number of patients according to ISS in the two groups separated by sex can be seen in Table 3 . 

**Figure 1.**
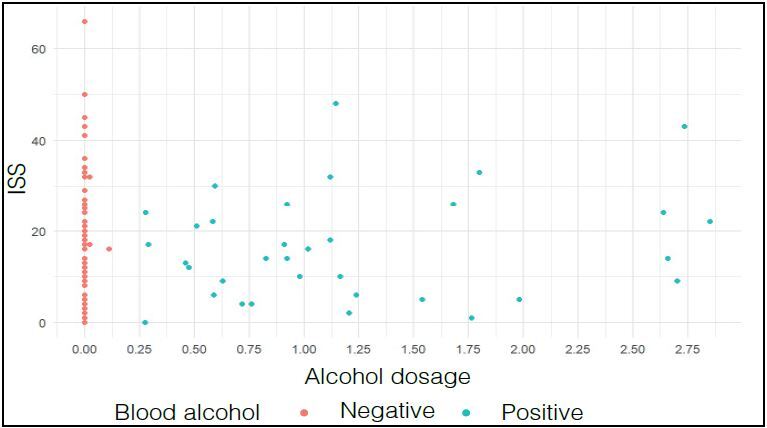
Relationship between Severity Index (ISS) and alcohol dosage of all patients enrolled in the study (p= 0.0803

 There was no correlation between the severity index (ISS) and alcohol dosage ( Figure 1 ). 

**Table 3.** Comparison of victims’ ISS value and blood alcohol concentration

	**Men**			**Women**		
	**ALL n (%)**	**POSITIVE n (%)**	**NEGATIVE n (%)**	**ALL n (%)**	**POSITIVE n (%)**	**NEGATIVE n (%)**
Mean ISS	15.11 (± 10.24)	16 (± 11.98)	14.84 (± 9.69)	17.33 (± 13.76)	22.5 (± 2.12)	17.02 (± 14.11)
Light ISS (0 -15 points)	80 (58.8%)	18 (22.5%)	62 (77.5%)	19 (52.8%)	0 (0%)	19 (100%)
Moderate ISS (16 -24 points)	33 (24.3%)	7 (21.2%)	26 (78.8%)	10 (27.8%)	2 (20.0%)	8 (80%)
Severe ISS (≥ 25 points)	23 (16.9%)	7 (30.4%)	16 (69.6%)	7 (19.4%)	0 (0%)	7 (100%)
Total	136	32 (23.5%)	104 (76.5%)	36	2 (5.6%)	34 (94.4%)

Caption: N: number of patients; %: percentage of patients; ± = standard deviation; ISS: *Injury Severity Score*

**Figure 2.**
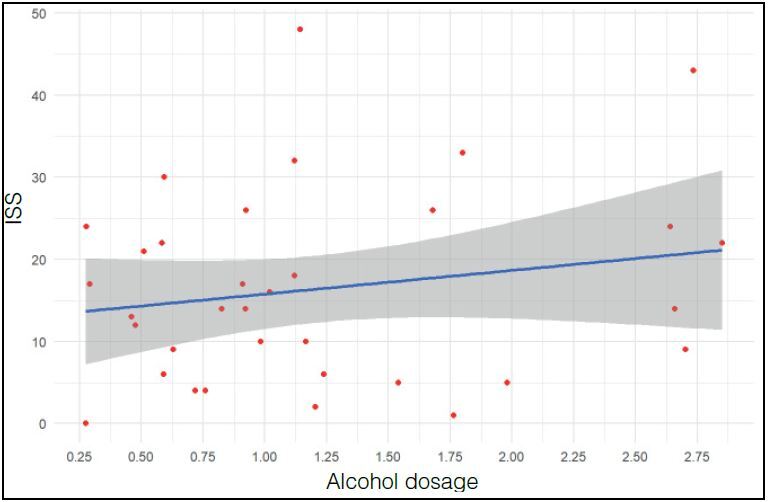
Relationship between Severity Index (ISS) and Alcohol Dosage of patients who had positive alcohol levels (≥ 0.2 g/L) (p= 0.1901)

 There was no correlation between ISS and blood alcohol in patients with alcoholic dosage > 0.2 g/L ( Figure 2 ) 

**Figure 3.**
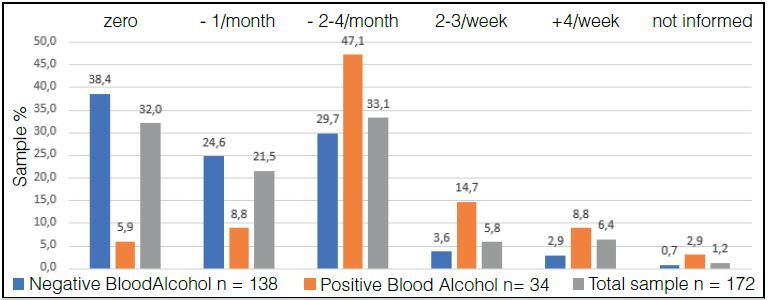
Percentage of patients in the Positive Alcohol, Negative Alcohol and Total Sample Groups distributed according to the frequency of alcohol intake (X² = 34.2368, p = 0.000).

 In the qualitative analysis, in relation to alcohol consumption, in response to question 1 “frequency of alcohol consumption”, 115 (66.8%) participants consume alcohol with varying frequencies, data seen in Figure 3 . The Positive Alcohol Group consumes alcohol more frequently than the Negative Alcohol Group.

**Figure 4.**
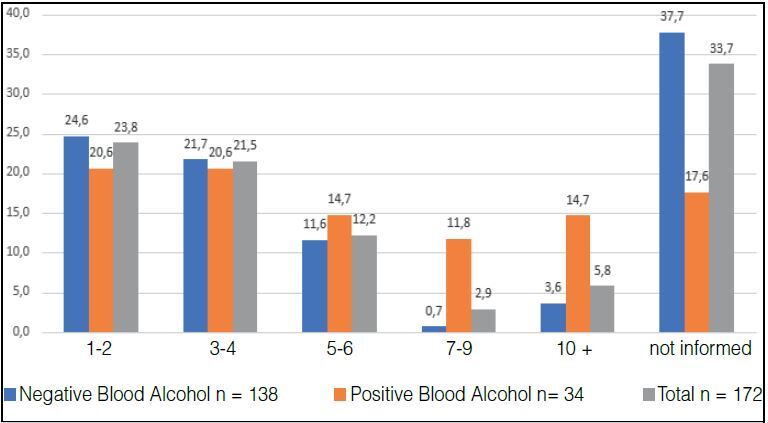
Percentages of patients in the Negative and Positive Alcohol and Total Sample Groups distributed according to the number of daily doses of alcoholic beverage ingested (X² = 13.6692 p= 0.0084).

 In the answer to question 2 “how many alcoholic drinks do you consume in a normal day?” The Positive Alcohol Group ingests more doses per day than the Negative Alcohol Group ( Figure 4 ). 

**Figure 5.**
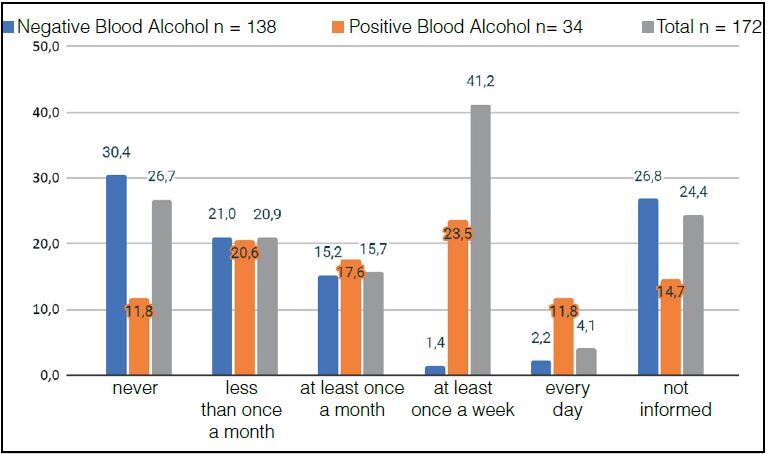
Percentages of patients in the Positive and Negative Alcohol and Total Sample Groups distributed according to the temporal frequency that drink four (women) or five (men) doses on a single occasion (X² = 19.7918, P = 0.0005).

 In answer to question 3 “how many times do you ingest four doses (women) or five doses (men) on a single occasion”. The Positive Alcohol Group showed a higher percentage of participants who drink abusively at least once a week or daily ( Figure 5 ). 

**Figure 6.**
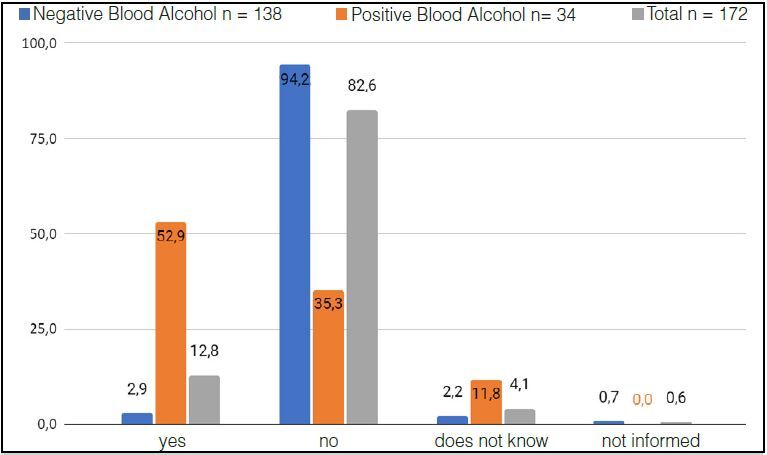
Percentages of participants in the Positive, Negative and Total Alcohol Group who drank or did not drink alcohol six hours before the accident (X²: 70.7286 p:0.000).

 In the answer to question 4 “did you drink alcohol in the six hours before the accident”. Most of the Positive Alcoholic Group had ingested some type of drink six hours before the accident, diverging from the Negative Alcoholic Group ( Figure 6 ). 

Table 4 shows the correlation between the different qualitative variables and the ISS level of all patients grouped by Total Group (n= 172), Positive Alcohol Group (n= 34) and Negative Alcohol Group (n= 138). The F test evaluated which variables are determinant in the severity of the accident. 

**Table 4.** Correlation between the different qualitative variables of the sample and the ISS value, separated by all patients in the sample, patients with positive blood alcohol and patients with negative blood alcohol.

	**F-value**			**Pr(> F)**		
	**All patients included**	**Patients with positive blood alcohol**	**Patients with negative blood alcohol**	**All patients included**	**Patients with positive blood alcohol**	**Patients with negative blood alcohol**
Day of the Week	0.453	0.35	0.31	0.84	0.89	0.92
Age group	0.324	1.07	0.68	0.86	0.37	0.60
Gender	1.142	0.57	1.02	0.28	0.45	0.31
Skin color	0.288	2.80	0.31	0.83	0.07	0.81
Education	1.824	1.48	1.12	0.11	0.23	0.34
Driver’s License	0.636	4.66	0.40	0.53	0.03	0.66
Victim	0.924	1.40	0.33	0.43	0.26	0.79
Vehicle	1.253	1.01	0.63	0.28	0.41	0.67
Type of Accident	1.576	0.29	2.30	0.14	0.91	0.03
Pre-hospital Care	5.841	0.76	6.90	0.00	0.47	0.00
Type of Service	3.109	0.86	3.83	0.01	0.50	0.00
Question 1	1.546	0.88	1.07	0.19	0.48	0.37
Question 2	1.653	1.07	2.61	0.15	0.39	0.02
Question 3	1.747	1.49	0.86	0.12	0.23	0.50
Question 4	4.600	10.10	0.05	0.01	0.00	0.94

Df: degree of freedom; Sum Sq: sum of squares; Mean Sq: mean squares; F value: F statistic; Pr(> F)= p-value for F statistic.

A significant correlation was observed in the Driver’s License variable for the Positive Group for alcohol, where patients who did not have a driver’s license had more severe lesions than those who did (mean ISS of 21.61 versus 13.14, respectively) (p= 0.03).

The variable Type of Accident had its significance level evidenced in patients in the Negative Group for alcohol (p= 0.03). The victims of lateral collision presented ISS= 17.4, a higher value compared to the other mechanisms (frontal collision 14.9; runover: 16.04; fall: 15.8; rollover: 13.5; multiple: 11.2; rear collision: 4.71).

Prehospital care was statistically relevant in the Total Group and Negative Alcohol Group (p= 0.00). In the Total Group of patients, 6 (3.5%) individuals were unable to answer if they had pre-hospital care, with ISS= 30.33, higher than the other victims (Yes= 15.36; No= 6.66). In the Negative Alcoholic Group, 3 patients were unable to report whether they had treatment, with ISS value = 29.33, higher than the others (Yes= 15.37; No= 7.2).

The variable Type of Care was also relevant in the Total Group and Negative Alcohol Group (p= 0.01 and p= 0.00, respectively). In the Total Group, 13 patients were unable to answer the question with ISS= 24.38, higher than the other patients (Águia= 16.90; Firefighters= 15.67; SAMU= 13.31; Others= 7.62).

Question 2 was significant in the Negative Alcohol Group (p= 0.02). A single patient reported drinking 7-9 doses, with the highest ISS value = 32; 34 patients reported consuming 1-2 doses (ISS= 17.26), 52 patients reported no consumption (ISS= 16.96), 5 reported consuming ≥ 10 doses (ISS= 16.6); 30 reported consuming 3-4 doses (ISS= 12.7); 16 patients consumed 5-6 doses (ISS= 9.8).

Question 4 showed a significant correlation in two groups, Total patients (p= 0.01) and Positive Alcohol (p= 0.00). In the Total Group, 22 patients reported “yes” and had ISS= 14.27; 142 answered “no” with ISS= 15.23; 7 answered “do not know” with ISS= 27.71; 1 patient “did not answer” with ISS= 8.0. In the Positive Group, 18 patients reported “yes” with ISS= 13.66; 12 answered “no” ISS= 13.83; 4 did not know how to answer (“do not know”) ISS= 36.25.

The variables mentioned, according to their groups, had a statistically significant relationship with the severity of the accident, measured by the ISS, at 95% confidence. For the other independent variables of the groups analyzed, it was possible to state that there are no statistically significant relationships with the level of severity of the injuries.

## DISCUSSION

 The association of alcohol with driving is one of the main factors related to traffic accidents with victims. ^1^ According to the American College of Surgeons, ^11^ preventable injuries are the main causes of chronic disabilities in Americans. ^11^ In Brazil, traumas are treated mainly in hospitals of the Unified Health System (SUS), and contribute significantly to the overcrowding of services, requiring a significant part of budgetary resources. ^12^

 This study evaluated the relationship between blood alcohol levels of victims of traffic accidents and the severity of injuries measured by the ISS. Previous studies show a relationship between the presence of alcohol and the risk of more serious injuries ^13^ , however, the blood alcohol dosage in these studies was performed post-mortem, and may not reflect the alcohol concentration at the time of the accident. ^9^ It is also noted that most studies evaluated mortality, without reference to morbidity. ^5^^-^^7^^,^^9^^,^^14^^,^^15^

 Blood alcohol dosage, when performed at the beginning of care, can help in the conduct of treatment (level of consciousness and hypotension). ^11^^,^^16^ Blood alcohol can increase vasodilation and impair volume resuscitation, in addition to greater predisposition to other complications. ^17^

 Studies have investigated the correlation between acute alcohol intoxication and the morbidity and mortality of traumatic injuries, however, there is no agreement in the literature and the results range from lower to higher chance of mortality and non-interference. ^6^^-^^8^^,^^14^^,^^15^^,^^17^^,^^18^ Stübig et al. ^6^ report higher mortality of patients with positive blood alcohol levels (4.6% vs. 2.2%). Ponce et al. ^13^ and Andreuccetti et al. ^4^ evaluated fatal victims of traffic accidents in the city of São Paulo and reported that more than 40% had positive blood alcohol levels. The evidence was also reported by other researchers. ^7^ Ahmed and Greenberg ^15^ maintain that the presence of alcohol does not alter length of stay and mortality rate. ^15^

 Stübig et al. ^6^ also states that the higher the blood alcohol level, the more severe the lesions. This relationship was not validated in our sample, as alcohol was not an aggravating factor for the injuries suffered by patients. 

 The predominance of young male adults has been recorded in many studies involving accidents of a traumatic nature ^15^^,^^18^^,^^19^^,^^20^ , which was also validated in this study. According to Chen et al. ^20^ age is an important factor in the involvement in traffic accidents. ^20^ The high-risk trend is concentrated in the age group from 18 to 30 years old. ^20^ However, our study does not validate this association, as we did not identify statistical significance between the severity of the injuries and the different age groups evaluated, whether or not associated with alcohol. 

 As in our sample, the lower involvement of women in traffic accidents is frequently reported. ^21^ The lower proportion of women in accidents and with alcohol present is associated with differences in the consumption habits and behavior of Brazilian women. ^13^ Women are more cautious and less aggressive than men in terms of driving attitude. However, when involved in accidents or under the influence of alcohol, they present more serious injuries. ^20^ Although we observed a difference in the severity of women in the different groups, this value was not significant. 

 The economic difficulty and the emergence of several delivery apps in the country contributed to the growth in the motorcycle fleet. ^12^ In other studies, motorcyclists had a higher chance of injury and death compared to drivers of other vehicles. ^15^ In general, they have a tendency to make impulsive decisions and have greater risk behavior, which is aggravated when drunk. ^5^^,^^6^^,^^15^

Although the motorcycle was the main vehicle involved in accidents in this sample, we did not identify statistical significance compared to other vehicles, presence of alcohol and severity of injuries, a finding which differs from other reports in the literature.

 In this study, the lack of driver’s license in the alcohol-positive population generated more serious injuries than in those who had a license. A lack of a driver’s license may indicate that the driver is unskilled or unmotivated to drive safely. ^9^

The severity of the injuries in our study was not linked to the presence of alcohol, but to other factors, such as the collision mechanisms.

 According to data from the Surveillance of Chronic Diseases by Telephone Survey (VIGITEL – 2013), ^22^ the habit of drinking is reported by one tenth of the Brazilian population, also being highly present in this sample. 

 In general, we did not find a significant difference in the correlation between the level of severity of the lesion and the alcohol levels of the patients. This finding corroborates some studies ^,15^ but is conflicting with others, ^6^ with no consensus among researchers. That said, alcohol alone cannot be considered a predictor of more serious injuries. 

The small sample size was one of the limitations found in this study. The loss due to insufficient data (in the medical records) and the exclusion of death cases lead to the decreased sample. In the future, similar analyses will be required, with larger samples. Other studies are necessary to evaluate the relationship between the severity of the injury associated with alcohol and other variables: time of accidents, weather conditions, use of protective equipment, purpose of the trip, fatigue, distraction and sleep deprivation. Continuous evaluation of the evidence is necessary to assess the effectiveness of the programs instituted.

This investigation was limited to the severe trauma population in a single hospital; thus, our inferences may not be generalizable to other populations or institutions, but we believe that the results presented here can serve as a starting point and support for the implementation of evidence-based public prevention policies.

## CONCLUSION

About 21% of the victims included in the sample had positive blood alcohol levels. Men with a mean age of 37.2 ± 14.75 years, motorcycle drivers and people with higher blood alcohol concentrations appeared in higher prevalence.

There was no correlation between the severity of injuries and blood alcohol levels of traffic accident victims admitted to a referral hospital. Taking into account our results, alcohol alone cannot be considered a predictor of more serious injuries.
